# Patient Satisfaction and Its Predictors With Perioperative Anesthesia Care at Two General Hospitals in Southwestern Saudi Arabia

**DOI:** 10.7759/cureus.33824

**Published:** 2023-01-16

**Authors:** Yahya M Alnashri, Omar Y Alfaqih, Malak A Buhaliyqh, Rwan A Mossery, Ibrahim R Alamri, Nujud A Mahfouz, Norah H Alsifsafi, Zahra A Alzubaidi, Suhail S Alfaifi, Abdulghani S Sadaqa, Saud M Alnashri, Faisal A Fallata, Mosad M Odah, Ashraf Ewis

**Affiliations:** 1 Medicine, Umm Al-Qura University, Al-Qunfudhah, SAU; 2 Medicine, Albaha University, Albaha, SAU; 3 Medicine, Jazan University, Jazan, SAU; 4 Faculty of Medicine, Umm Al-Qura University, Al-Qunfudhah, SAU; 5 Medicine, Umm Al-Qura University, Makkah, SAU; 6 Surgery, Al-Qunfudah General Hospital, Al-Qunfudhah, SAU; 7 Internal Medicine, Al-Qunfudah General Hospital, Makkah, SAU; 8 Biochemistry, Umm Al-Qura University, Al-Qunfudhah, SAU; 9 Biochemistry, Benha University, Benha, EGY; 10 Public Health, Faculty of Health Sciences, Umm Al-Qura University, Makkah, SAU; 11 Public Health and Occupational Medicine, Minia University, El-Minia, EGY

**Keywords:** anesthetic care, perioperative care, leiden perioperative care patient satisfaction questionnaire (lppsq), saudi arabia, anesthesia, patient satisfaction

## Abstract

Introduction

Patient satisfaction is one of the most crucial quality assessment and improvement indicators in anesthesia. Different factors reflect satisfaction such as postoperative pain, procedure duration, patient-physician relationship, inpatient services, and waiting time. A high level of satisfaction can lead to better outcomes in many ways, such as decreasing future surgeries fear and strengthening the healthcare system trust among the population. Therefore, this study aimed to evaluate the satisfaction level and its predictors with perioperative anesthesia care among patients subjected to different surgeries in two general hospitals in southwestern Saudi Arabia.

Methodology

A cross-sectional study was conducted among patients admitted to different surgical specialties at two general hospitals in Al-Qunfudhah governorate in October 2022. Data were collected through interviews with postoperative patients and checking their medical data from the patient's medical reports. However, all surgical patients aged more than 18 consider as inclusion. In contrast, intensive care unit (ICU) admission, local anesthesia, refusal to participate, and cognitive and communication impairment are the exclusion. Perioperative patient satisfaction was assessed using the Leiden Perioperative Care Patient Satisfaction Questionnaire (LPPSq).

Results

Eighty-three of 201 patients were included in the final analysis. The overall level of patient satisfaction concerning perioperative anesthetic care was calculated to be 73.5%. Hospital setting, admission type, BMI, and smoking were statistically associated with perioperative anesthesia patient satisfaction. Additionally, the most frequently reported unpleasant anesthetic side effect was shivering, followed by postoperative pain at a frequency of 42 (50.6%) and 37 (44.6%), respectively.

Conclusion

A moderate level of patient satisfaction concerning perioperative anesthetic care was detected. Smoking, BMI, admission type, and hospital setting were significantly associated predictors for patients' satisfaction. In order to present a complete picture, we recommend that future research concentrate on additional elements of patient satisfaction, particularly operating room turnover and standards for discharge. Additionally, we propose a routine evaluation before patients' discharge when patients are altering and oriented. Periodic evaluation and enhancement of patient satisfaction with perioperative anesthetic care should be employed and promoted.

## Introduction

Patient satisfaction is a hot issue in contemporary medicine and a widely recognized indicator of healthcare quality [1]. Patient satisfaction is a complicated concept that depends heavily on the patient's subjective evaluation [2]. It refers to the extent to which patients' expectations are met by the treatment offered [3]. Patient satisfaction is essential in assessing the quality of care, and it remains the most accurate approach to evaluating the outcome from the patient's perspective [4]. Patient satisfaction affects a range of patient behaviors, such as healthcare resource consumption, treatment adherence, and the constancy of their connection with practitioners [5,6].

Patient satisfaction offers patient-centered care in an environment that treats patients for whom they are and where they are in their life cycle [7]. By meeting their requirements at that time, in harmony with the health system's goal of caring for patients' bodies, minds, and spirits, as well as positively impacting personnel, the community, and the organization's health. It is also tied to many other aspects of the patient, including the patient's psychological, social, cultural, and values status, previous experiences, and expectations for the future [8,9]. Nevertheless, the accessibility and convenience of services that rely on institutional regulations, interpersonal interactions, and the competency of health professionals are all significant determinants [10].

Perioperative anesthesia care considers a vital aspect of medical care. Several types of anesthesia, such as regional anesthesia, general anesthesia, and a possible mix of regional and general anesthetic, are commonly employed in today's surgery. Anesthesiologists play a significant role in preoperative evaluation to establish risk factors associated with anesthesia and surgery, intraoperative management, and postoperative adverse effect therapy. Consequently, a complete evaluation of patient satisfaction following anesthesia care is a crucial metric for quality control and continual improvement in hospital services [11]. Although many perioperative factors, such as communication skills with the patient, adequacy of information provided during the preoperative period, compassion toward the patient while they are experiencing stress, type of anesthesia, perioperative nausea and vomiting, intraoperative awareness, the length of surgery, and postoperative pain are among the influences of perioperative patient satisfaction [8,9,12]. Furthermore, patient dissatisfaction can be detrimental to health practitioners and facilities [13]. For every unsatisfied patient who expressed unhappiness with the experience, many more will remain quiet and will likely never return to the facility, while others will tell their relatives and friends about their poor experiences [14].

Australian research evaluated patient satisfaction and other specified outcomes, including nausea, vomiting, and pain. Overall patient satisfaction was (96.8%), with 2.3% of patients somewhat dissatisfied and 0.9% dissatisfied with their anesthesia service [15]. In a study of Japanese patients, 3.9% were unsatisfied with the anesthesia. Most unsatisfied patients were between the ages of 20 and 39, were female, and underwent spinal anesthesia rather than general anesthesia [16].

Possessing adequate patient satisfaction data allows a facility to enhance and comprehend its strengths, as well as to target areas where performance is insufficient, and to give a chance for the repair or modification of the discovered gaps. Overall, assessing anesthesia satisfaction and paying attention to it will expand our knowledge and address a knowledge gap in Saudi Arabia that has yet to be filled to the best of our knowledge. This study aims to evaluate the level of patient satisfaction with perioperative anesthesia care and its predictors among patients admitted and subjected to different operative procedures in different surgical wards at two general hospitals in Al-Qunfudah governorate, southwestern Saudi Arabia.

## Materials and methods

A cross-sectional study was done in Al-Qunfudhah governorate, southwestern Saudi Arabia. This study was conducted at two governmental general hospitals - Al-Qunfudhah General Hospital and South Al-Qunfudhah General Hospital. This study included all surgical patients across the main different surgical specialties from October 1, 2022 to October 31, 2022. The inclusion criteria for this study included surgical patients 18 years of age or older. Patients under 18, those who declined to participate, those who underwent surgery under local anesthesia, patients with cognitive impairment and communication issues, and patients admitted to the Intensive Care Unit (ICU) were excluded from this study.

Before the study was conducted, ethical approval was obtained from the biomedical research ethics committee at Umm Al-Qura University (HAPO-02-K-012-2022-08-1163). To ease the researchers' work at the two hospitals, the Directorate of Health Affairs in Al-Qunfudhah granted additional approval and official consent to conduct the study.

To verify the absence of any bias throughout the data-gathering process, all data collectors were medical interns from non-surgical departments. The data collectors engaged in a series of meetings to guarantee that the questionnaire would be applied equally to all participants. In addition, the authors audited the data collection process to ensure the consistency and accuracy of the data.

Data collectors interviewed patients post-operatively in the surgical ward and whenever needed some information was extracted from the patients' medical records. Before starting the interview with the patients, the data collectors explained to each participant that their responses will not interfere with their care plan or affect their hospital stay. In addition, the data collectors guaranteed the patients' ability to decline participation or withdraw at any point in time. Then, they received their signed informed consent for going through the interview. Finally, they collect the questions that must be extracted from each patient's medical record.

The main research question of this study was the evaluation of perioperative patient satisfaction with anesthetic care. This was assessed using the Leiden Perioperative Care Patient Satisfaction Questionnaire (LPPSq). The LPPSq is a good instrument for measuring perioperative patient satisfaction following anesthesia care [17]. Its 21 questions are suited for research purposes (items of information, fear, concern, and the staff-patient relationship with 4, 4, and 13 questions each, respectively). LPPSq is a 5-point Likert scale used to quantify the level of satisfaction: 1, strongly satisfied; 2, satisfied; 3, neither satisfied nor dissatisfied; 4, dissatisfied; and 5, strongly dissatisfied. During analysis, respondents who were strongly satisfied and satisfied were categorized as satisfied, while respondents who were neither satisfied nor dissatisfied, dissatisfied, and strongly dissatisfied were categorized as dissatisfied.

Before conducting the main study, a pilot study was conducted on 20 patients from the general surgery and obstetrics departments to examine the clarity of questions and internal consistency of the questions as well as the inter-observer consistency of the data collectors. These pilot study collected data were not included in the main study.

The independent factors, however, evaluated sociodemographic data and other related patient's medical information. Specifically, hospital setting, gender, age, nationality, height, weight, education, marital status, occupation, smoking, type of admission, surgical specialty, anesthesia type, prior surgery with anesthesia, and preoperative and postoperative anesthesiologist visits. The American Society of Anesthesiologists (ASA) physical status classification system also was recorded for each patient. Before anesthesia, ASA aims to evaluate the patient's medical comorbidities and assists in identifying perioperative risks. It classified patients from one to six, with class one representing a normal healthy patient ranging to class six with brain-dead patients [18]. In addition to inquiring about the occurrence of any unfavorable anesthesia side effects, including postoperative pain, sore throat, nausea or vomiting, shivering, and hoarseness of voice.

Statistical analysis

Data were entered into the statistical package for social sciences (SPSS) software, (Version 29) for analysis. The body mass index (BMI) was computed by dividing the patient's weight in kilograms (kg) by the square of their height in meters (m^2^). Qualitative data were presented as frequency distribution (numbers and percentages), while quantitative data were determined using descriptive statistics (means and standard deviations). The only missing data were the height of one patient, which was substituted by the mean. The Shapiro-Will and Kolmogorov-Smirnov normality tests showed that the data were normally distributed. A binary logistic regression analysis was utilized to predict the association between patient satisfaction and several independent variables. Multicollinearity was also examined using the variance inflation factor, and it was determined that no multicollinearity existed. The Hosmer-Lemeshow test was utilized to evaluate model fitness. The odds ratio was calculated with a 95% confidence interval. P-values of 0.05 and less were regarded as statistically significant.

## Results

**Figure 1:**
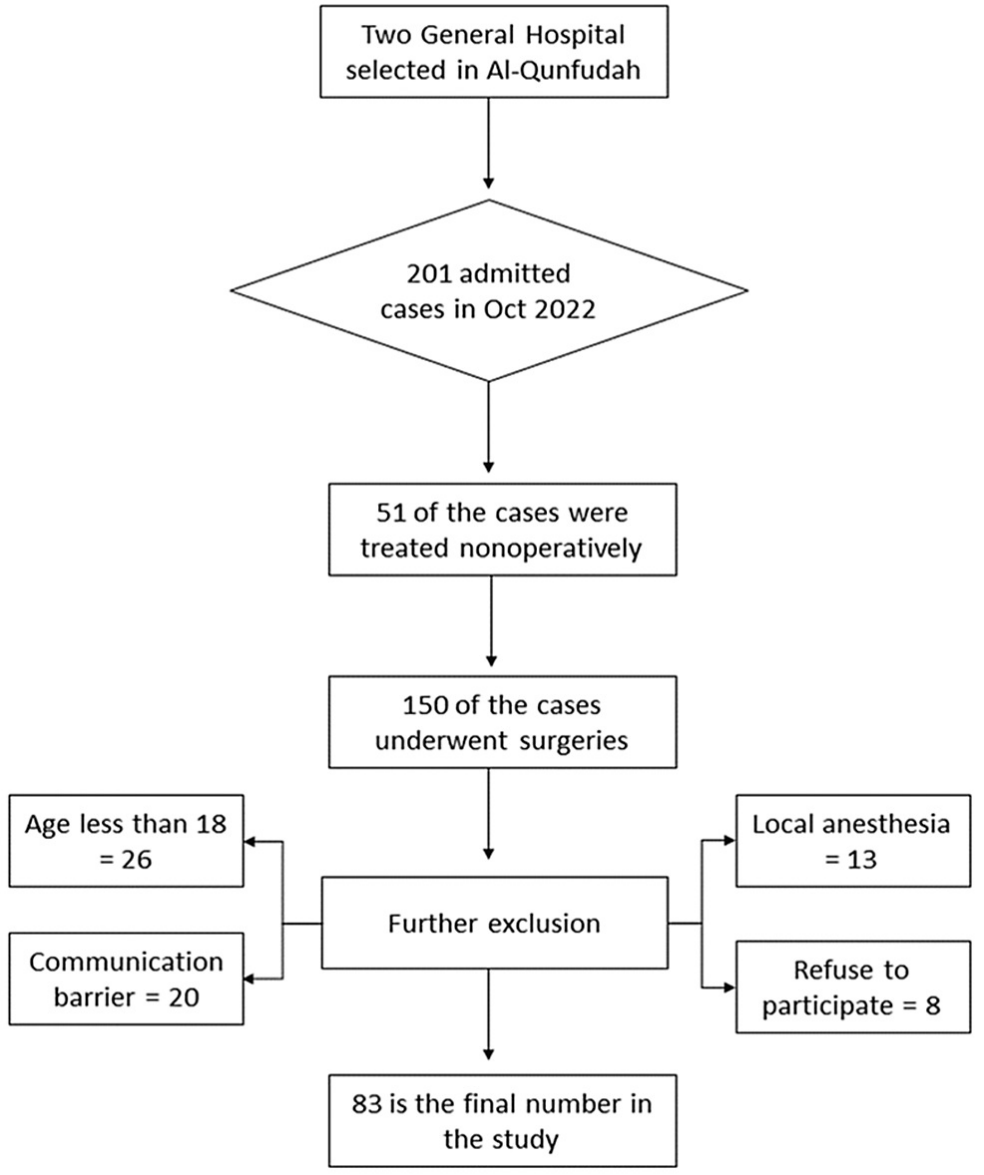
The number of patients eligible to participate in the study, patients who were excluded, and those who were included in the final analysis.

Eighty-three of the 201 patients admitted to the surgical ward met the inclusion criteria and were included in the final analysis (Figure 1). The male-to-female ratio was 1:5.9, with 12 men and 71 women participating. The mean age was 32.75 years, with a standard deviation of 7.75 years and a range of (19-53) years. The mean BMI was 28.8, with a standard deviation of 6.2. The majority of the study's participants were married n=72 (86.7%) and unemployed n=65 (78.3%) (Table 1). Regarding the surgical specialty, n=59 (71.1%) cases were admitted under the obstetrics and gynecology department. The preponderance of cases in this study were emergency patients, n=56 (67.5%). The most frequently reported unpleasant anesthetic side effect is shivering, followed by postoperative pain at a frequency of n=42 (50.6%) and n=37 (44.6%), respectively (Table 2).

**Table 1:** Sociodemographic information of the studied patients who underwent surgery at two General Hospitals in Al-Qunfudhah governorate, October 2022.

Variable	Category	N=83	%
Gender	Female	71	85.5
	Male	12	14.5
Nationality	Saudi	72	86.7
	Non-Saudi	11	13.3
Marital status	Married	72	86.7
	Single	11	13.3
Education	High school or below	42	50.6
	Bachelor or higher	41	49.4
Occupation	Employed	18	21.7
	Unemployed	65	78.3
Hospital setting	Al-Qunfudhah General Hospital	38	45.8
	South Al-Qunfudhah General Hospital	45	54.2
Smoking	Yes	9	10.8
	No	74	89.2
American Society of Anesthesiologists (ASA)	I	13	15.7
	II	65	78.3
	III	5	6.0

**Table 2:** Distribution of intraoperative factors of surgical inpatients who underwent surgery, as well as postoperative pain and adverse outcomes in two general hospitals, Al-Qunfudhah governorate, October 2022.

Variable	Category	N	%
Preoperative visits by an anesthesiologist	Yes	79	95.2
	No	4	4.8
Postoperative visits by an anesthesiologist	Yes	46	55.4
	No	37	44.6
Admission Type	Elective	27	32.5
	Emergency	56	67.5
Surgical specialty	Obstetrics and Gynecology	59	71.1
	General surgery	24	28.9
Type of anesthesia	General	39	47.0
	Spinal	44	53.0
Duration of anesthesia	< 60 minutes	33	39.8
	60 – 120 minutes	17	20.4
	More than two hours	33	39.8
Prior surgery with anesthesia	Yes	46	55.4
	No	37	44.6
Postoperative pain	Yes	37	44.6
	No	46	55.4
Sore throat	Yes	24	28.9
	No	59	71.1
Nausea and vomiting	Yes	31	37.3
	No	52	62.7
Shivering	Yes	42	50.6
	No	41	49.4
Hoarseness	Yes	27	32.5
	No	56	67.5

**Figure 2:**
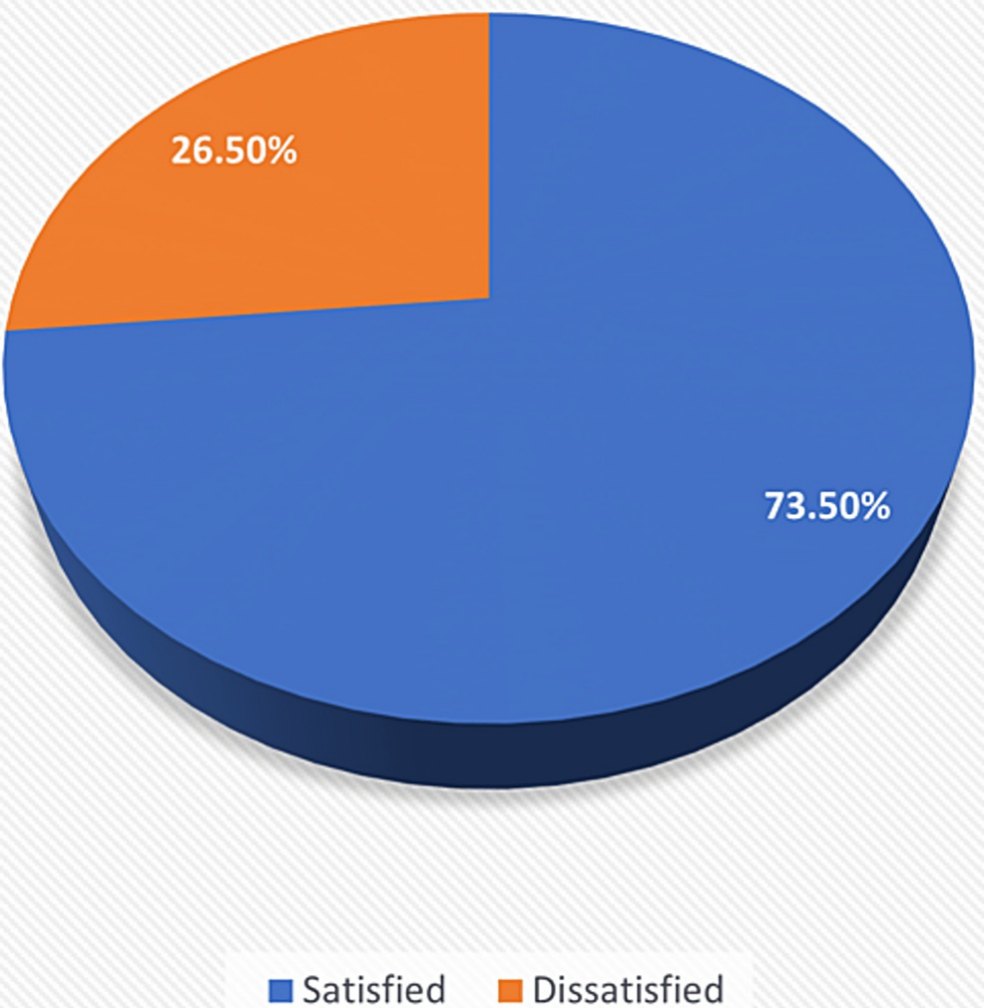
Percentage of patient satisfaction with perioperative anesthesia care at the studied two general hospitals in Al-Qunfudhah governorate.

The overall level of patient satisfaction concerning perioperative anesthetic care was 73.5% (Figure 2). The percentage of satisfied patients was higher in males compared to females, and in those who had a bachelor's degree or higher, non-Saudi residents, married, employed, those who had undergone prior surgery, seen by anesthesiology pre- and post-operatively, who had general surgery operations, who received general anesthesia, and whose procedure lasted for less than an hour. However, these differences were not statistically significant (Table 3).

**Table 3:** Binary logistic regression analysis of the association between perioperative patient satisfaction and other independent variables among surgical patients in Al-Qunfudhah governorate, October 2022.

Variable	Category	Satisfaction		P value	OR	95% C.I. for OR	
		Satisfied (%)	Dissatisfied (%)			Lower	Upper
Gender	Female	52 (73.2)	19 (26.8)	1	1	1	1
	Male	9 (75)	3 (25)	0.796	1.829	0.019	176.766
Nationality	Saudi	52 (72.2)	20 (27.8)	1	1	1	1
	Non-Saudi	9 (81.8)	2 (18.2)	0.269	5.552	0.267	115.650
Marital status	Married	53 (73.6)	19 (26.4)	1	1	1	1
	Single	8 (72.7)	3 (27.3)	0.535	0.277	0.005	15.897
Education	Bachelor or higher	33 (80.5)	8 (19.5)	0.441	0.485	0.077	3.053
	High school or below	28 (66.7)	14 (33.3)	1	1	1	1
Occupation	Employed	16 (88.9)	2 (11.1)	1	1	1	1
	Unemployed	45 (69.2)	20 (30.8)	0.841	0.767	0.058	10.152
Hospital setting	South Al-Qunfudhah General Hospital	29 (64.4)	16 (35.6)	1	1	1	1
	Al-Qunfudhah General hospital	32 (84.2)	6 (15.7)	0.029	11.679	1.293	105.514
Smoking	No	57 (77)	17 (23)	1	1	1	1
	Yes	4 (44.4)	5 (55.6)	0.029	0.011	0.000	0.625
American Society of Anesthesiologists (ASA)	I	10 (76.9)	3 (23.1)	1	1	1	1
	II	48 (73.8)	17 (26.2)	0.503	0.294	0.008	10.579
	III	3 (60)	2 (40)	0.110	0.022	0.000	2.368
Preoperative visits by an anesthesiologist	Yes	59 (74.7)	20 (25.3)	1	1	1	1
	No	2 (50)	2 (50)	0.525	0.287	0.006	13.361
Postoperative visits by an anesthesiologist	Yes	23 (85.2)	4 (14.8)	1	1	1	1
	No	38 (67.9)	18 (32.1)	0.106	0.237	0.042	1.356
Admission Type	Elective	19 (70.4)	8 (29.6)	1	1	1	1
	Emergency	42 (75)	14 (25)	0.039	8.864	1.114	70.518
Surgery specialty	Obstetrics and Gynecology	42 (71.2)	17 (28.8)	1	1	1	1
	General surgery	19 (79.2)	5 (20.8)	0.347	10.638	0.077	1469.406
Type of anesthesia	General	31 (79.5)	8 (20.5)	1	1	1	1
	Spinal	30 (68.2)	14 (31.8)	0.432	2.281	0.292	17.826
Duration of anesthesia	Less than 60 minutes	26 (78.8)	7 (21.2)	1	1	1	1
	60 – 120 minutes	12 (70.6)	5 (29.4)	0.835	0.792	0.088	7.091
	More than 120 minutes	23 (69.7)	10 (30.3)	0.289	0.310	0.036	2.703
Prior surgery (with anesthesia)	No	26 (70.3)	11 (29.7)	1	1	1	1
	Yes	35 (76.1)	11 (23.9)	0.140	3.968	0.635	24.777
Postoperative pain	No	31 (67.4)	15 (32.6)	1	1	1	1
	Yes	30 (81.1)	7 (18.9)	0.261	0.296	0.035	2.471
Postoperative sore throat	No	42 (71.2)	17 (28.8)	1	1	1	1
	Yes	19 (79.2)	5 (20.8)	0.926	1.129	0.086	14.775
Postoperative nausea and vomiting	No	35 (67.3)	17 (32.7)	1	1	1	1
	Yes	26 (83.9)	5 (16.1)	0.309	2.755	0.390	19.454
Postoperative shivering	No	30 (73.2)	11 (26.8)	1	1	1	1
	Yes	31 (73.8)	11 (26.2)	0.078	0.144	0.017	1.246
Postoperative hoarseness	No	38 (67.9)	18 (32.1)	1	1	1	1
	Yes	23 (85.2)	4 (14.8)	0.106	0.267	0.042	1.356

In a binary logistic regression model, hospital setting, admission type, BMI, and smoking were statistically associated with perioperative patient satisfaction with anesthesia care. The odds of patient satisfaction at Al-Qunfudhah General Hospital were 1.3 times more than at South Al-Qunfudhah General Hospital. In emergency procedures, the odds of patient satisfaction were 8.9 times greater than in elective surgeries. The higher BMI of the patient was associated with a greater level of satisfaction.

## Discussion

Patient satisfaction is the equilibrium between past expectations and subsequent judgments of the quality of health care received; thus, poor quality will discourage patients from using the service as they should [3]. Any complaints or issues highlighted by patients must be taken into account through a trail of investigation, analysis, and, subsequently, implementation of relevant solutions [19].

In the present study, the rate of patient satisfaction was 73.5% at two general hospitals in the Al-Qunfudhah governorate. In Saudi Arabia, however, a lower patient satisfaction rate was reported according to a multicentric study conducted in different cities in the country in 2018, which revealed that 56.5% of patients were satisfied [8]. A possible explanation for the higher satisfaction rate in our sample may be due to the implementation of the health sector transformation program in recent years. The program aims to restructure the health sector to be a comprehensive, effective, and integrated system with a domain that focuses on the satisfaction of the beneficiaries. Another likely reason for the discrepancy is that the previously mentioned study used another assessment tool to measure patient satisfaction.

Exploring the level of satisfaction in different nations, an Ethiopian study conducted in 2022 indicated a lower satisfaction rating (64%) than our study [20]. In addition, a further study done at a comprehensive, specialized hospital in the north-central region of Ethiopia in 2021 indicated a satisfaction rate of 64.6% [21]. In 2020, a similar rate of 60% was recorded in an Ethiopian academic hospital [22]. On the same continent, An Eritrean study conducted in 2019 found that 68.8% of patients were satisfied with anesthesia care [9]. In addition, Egyptian research conducted in 2013 revealed a satisfaction rate of 61.9% [23]. The consistency between these research could be attributed to the employment of the same assessment tool (LPPSq) and the similar sociodemographic features and economic status of these countries. To further explain the causes of the dissatisfaction in the described research above, a large proportion accounted for postoperative nausea, vomiting, and pain [20,22].

In contrast, several studies have documented higher levels of satisfaction. According to a Canadian survey, the level of satisfaction is 98.8% [24]. Similarly, research in Australia discovered that 96.8% of patients were satisfied with anesthetic care [15]. In addition, a Japanese survey revealed that only 3.9% of participants were unsatisfied [16]. However, the cited studies do not align with the current research, which could be due to a variety of factors. First, the measurement tools employed in these investigations are different in each study. The second explanation may entail sociodemographic factors, hospital community awareness, organizational and structural setup, and a standardized preoperative and postoperative evaluation. And finally, the patient's subjective opinions.

Nonetheless, these high scores may provide a misleading picture since some patients may be influenced by the concern of receiving lesser care if they provide negative feedback. However, in our setting, we initially reassured patients that this would not impact their care plans. Second, all of the patients in our sample were admitted to government hospitals, which provide mandatory and equal care.

Interestingly, even with many similarities in the governorate, Al-Qunfudhah General Hospital was more significantly associated with satisfaction than South Al-Qunfudhah General Hospital. We could postulate this unpredictable difference that Al-Qunfudhah General hospital is older than south Al-Qunfudhah General Hospital. The staff and the quality control have more experience with patients' needs, expectations, and cultural backgrounds. Additionally, Al-Qunfudhah General Hospital's infrastructure and facility have just been renovated and opened in the past year, which undoubtedly provides a comfortable environment for patients.

In the present study, a higher level of education and the male gender were related to greater satisfaction levels; nevertheless, these associations did not achieve statistical significance. In conformity with this, a study discovered that satisfaction varies by gender, with ladies being less satisfied than males [16,25]. However, according to a different survey, ladies are more satisfied than males [26]. Patient satisfaction may be a subjective and complicated concept with respect to these differences. In keeping with our findings, an Indian study discovered that people with a higher degree of education are more satisfied with anesthesia services [27]. Some investigations hypothesize that this is because educated individuals are more capable of understanding and comprehending the nature of anesthesia procedures.

This study discovered a significant correlation between smoking and dissatisfaction. Most smoker patients, especially those who have been smoking for a long time, have multiple comorbid conditions, including issues with cardiovascular, respiratory, nervous system, and ability to heal wounds [28]. Also, smokers are encouraged to quit before surgery, subjecting them to nicotine withdrawal during this time. These elements raise the possibility of postoperative complications, which would have a detrimental effect on patient satisfaction.

Interestingly, patients who underwent emergency surgery were more satisfied than those of elective and showed statistical significance. This finding is consistent with an Eritrean study [9]. In that study, emergency surgery patients were more satisfied with perioperative anesthetic care than elective surgery patients. In contrast to these findings, an Indian study found a higher proportion of patient satisfaction among those who underwent elective surgery [27]. In emergency surgeries, patients may be more satisfied than other patients because the majority of them require fast treatment for their pain and life-threatening diseases. Consequently, alleviating their pain and condition during that period will constitute a substantial portion of their experience, and they may even neglect other parts of care. Besides this, in emergency cases, healthcare practitioners are well-prepared and prioritize the treatment of life-threatening conditions over non-emergency conditions.

In our study, patients who had postoperative visits were more satisfied than those who did not. This discovery is comparable to that discovered in Switzerland [29]. The rationale behind postoperative visits is that they strengthen the relationship between the patient and anesthesiologist and allow the patient to express their concerns and experiences. Additionally, it boosts the chances of enhanced postoperative treatment.

It is intriguing to note that there is a significant association between patient satisfaction and BMI, with higher BMI being associated with higher patient satisfaction. This may be because anesthesiologists pay more attention to patients who are overweight and obese. In terms of preoperative optimization, airway management, and postoperative pain control, these patients typically require greater levels of care.

This study, however, possesses some limitations. The main limitation is the small sample size. As it is not a multi-center study and just two general hospitals were recruited, it may impact the generalizability of the findings. Another limitation is that patients discharged during the first 24 hours of operation were not surveyed. Furthermore, a significant constraint due to the conceptual difficulties with patient satisfaction is that data were obtained before the participants were discharged, which may have prevented them from expressing their opinions due to their reliance upon the provided medical care. Finally, we cannot infer a temporal relationship between patient satisfaction and other independent factors due to the cross-sectional design of the study.

## Conclusions

The study concluded that nearly three quarters of patients were satisfied with perioperative anesthesia care. Smoking, BMI, hospital setting, and admission type were statistically associated with the perioperative anesthesia patient satisfaction. This study revealed that the level of patient satisfaction concerning perioperative anesthesia care was higher in males than females. On the other hand, this study identified some variables that interfere with patient satisfaction but are not statistically significant. Variables include patients seen by an anesthesiologist pre and postoperatively, patients who had a bachelor's degree or higher, patients who had undergone prior surgery, and procedures that lasted less than an hour. In order to provide a complete picture, we suggest that, in future research, patients should be explored for the reasons of dissatisfaction, and other components of patient satisfaction, such as operating room turnover and discharge criteria, should also be evaluated. We also recommend a routine evaluation before discharge, when patients have recovered from anesthesia, to distinguish surgical from anesthesia-related complications. In addition, a regular study and intervention should be conducted periodically to improve patient satisfaction.
